# A Comparison of CVR Magnitude and Delay Assessed at 1.5 and 3T in Patients With Cerebral Small Vessel Disease

**DOI:** 10.3389/fphys.2021.644837

**Published:** 2021-06-02

**Authors:** Michael S. Stringer, Gordon W. Blair, Yulu Shi, Iona Hamilton, David A. Dickie, Fergus N. Doubal, Ian M. Marshall, Michael J. Thrippleton, Joanna M. Wardlaw

**Affiliations:** ^1^Centre for Clinical Brain Sciences, University of Edinburgh, Edinburgh, United Kingdom; ^2^UK Dementia Research Institute at the University of Edinburgh, Edinburgh, United Kingdom; ^3^Beijing Tiantan Hospital Affiliated to Capital Medical University, Beijing, China; ^4^College of Medical, Veterinary, and Life Sciences, University of Glasgow, Glasgow, United Kingdom

**Keywords:** cerebrovascular reactivity, small vessel disease, magnetic resonance imaging, stroke, BOLD signal

## Abstract

**Background:**

Cerebrovascular reactivity (CVR) measures blood flow change in response to a vasoactive stimulus. Impairment is associated with several neurological conditions and can be measured using blood oxygen level-dependent (BOLD) magnetic resonance imaging (MRI). Field strength affects the BOLD signal, but the effect on CVR is unquantified in patient populations.

**Methods:**

We recruited patients with minor ischemic stroke and assessed CVR magnitude and delay time at 3 and 1.5 Tesla using BOLD MRI during a hypercapnic challenge. We assessed subcortical gray (GM) and white matter (WM) differences using Wilcoxon signed rank tests and scatterplots. Additionally, we explored associations with demographic factors, WM hyperintensity burden, and small vessel disease score.

**Results:**

Eighteen of twenty patients provided usable data. At 3T vs. 1.5T: mean CVR magnitude showed less variance (WM 3T: 0.062 ± 0.018%/mmHg, range 0.035, 0.093; 1.5T: 0.057 ± 0.024%/mmHg, range 0.016, 0.094) but was not systematically higher (Wilcoxon signal rank tests, WM: *r* = −0.33, confidence interval (CI): −0.013, 0.003, *p* = 0.167); delay showed similar variance (WM 3T: 40 ± 12 s, range: 12, 56; 1.5T: 31 ± 13 s, range 6, 50) and was shorter in GM (*r* = 0.33, CI: −2, 9, *p* = 0.164) and longer in WM (*r* = −0.59, CI: −16, −2, *p* = 0.010). Patients with higher disease severity tended to have lower CVR at 1.5 and 3T.

**Conclusion:**

Mean CVR magnitude at 3T was similar to 1.5T but showed less variance. GM/WM delay differences may be affected by low signal-to-noise ratio among other factors. Although 3T may reduce variance in CVR magnitude, CVR is readily assessable at 1.5T and reveals comparable associations and trends with disease severity.

## Introduction

Cerebrovascular reactivity (CVR) quantifies the change in blood flow in response to a vasoactive stimulus, providing an indicator of vascular health. CVR magnitude measures the amplitude of the response. However, CVR delay, the time between stimulus onset and observing a change in the blood flow, should also be considered to avoid underestimating CVR magnitude as response time can vary in different tissues (van Niftrik et al., 2017).

Impaired CVR is associated with several conditions, including cerebral small vessel disease (SVD), a major cause of stroke and dementia with no current treatments (Wardlaw et al., 2019). CVR magnitude is lower in patients with more severe SVD features on magnetic resonance imaging (MRI) (Blair et al., 2020), and CVR delay is longer in SVD patients than healthy controls (Sam et al., 2016; Thrippleton et al., 2018; Atwi et al., 2019). Therefore, exploring the role of CVR in SVD may provide valuable insight into disease progression mechanisms.

CVR is typically assessed using blood oxygenation level dependent (BOLD) MRI, and several different vasoactive stimuli are available (Liu et al., 2019). Common vasoactive stimuli include fixed or targeted CO_2_ inhalation, breath hold, and injection of acetazolamide. Fixed inhalation provides a robust stimulus applicable in many patient populations, including SVD (Thrippleton et al., 2018), the effectiveness of which is less dependent on patient compliance than breath hold and requires less specialist input than targeted inhalation.

Although field strength influences both the intra- and extravascular components of the BOLD signal (Silvennoinen et al., 2003), CVR comparisons at different field strengths remain limited (Driver et al., 2010; Triantafyllou et al., 2011; Peng et al., 2020). Increased field strength enhances contrast-to-noise ratio of the BOLD signal (Gati et al., 1997; Krüger et al., 2001; Triantafyllou et al., 2011) but increases susceptibility artifact (Bernstein et al., 2006). Additionally, CVR has mainly been measured at 3T or higher despite 1.5T MR scanners being more common clinically; therefore, the effect of field strength on CVR measurements in patient populations should be considered (Blair et al., 2016). Few studies have quantified the effect of field strength on CVR measurement at 1.5T and 3T. Triantafyllou et al. (2011) found the change in R2^∗^ per unit fractional flow change was 1.76 times higher at 3T than 1.5T using a fixed inhalation paradigm. Similarly, Peng et al. (2020) found a higher percentage signal change at 3T than 1.5T using breath hold. However, both studies were in healthy controls, and to our knowledge, no comparisons have compared CVR values at different field strengths in clinical populations.

We assessed subcortical gray (GM) and white matter (WM) CVR in patients with minor ischemic stroke at 1.5T and 3T to compare CVR magnitude and delay. Additionally, we compared associations between CVR and key clinical variables at each field strength.

## Materials and Methods

### Participants

We prospectively recruited patients with symptomatic minor, non-disabling, ischemic stroke as previously described (Wardlaw et al., 2009; Heye et al., 2016). We diagnosed patients based on clinical presentation, appearance on diffusion-weighted imaging and other relevant diagnostic MRI sequences as described in the STRIVE guidelines (Wardlaw et al., 2013). The patients had recovered from the minor stroke and were living independently in the community although many still had minor symptoms of the stroke. Many patients with stroke also have vascular risk factors, such as hypertension or hyperlipidemia; often have features of SVD on imaging, indicating chronic vascular brain damage; and are prescribed secondary prevention for stroke, including antihypertensive agents and lipid-lowering and antiplatelet drugs. Features of SVD on brain imaging commonly include WM hyperintensities (WMH), lacunes (small holes in subcortical GM or WM), microbleeds, and visible perivascular spaces (PVS). We excluded patients with disabling stroke (requiring assistance for activities in daily life), poorly controlled diabetes mellitus, hypertension, any psychiatric illness with the potential to limit study compliance, a family history of intracranial aneurysm, subarachnoid hemorrhage, known arteriovenous malformation, or contraindications to MRI.

We acquired written informed consent from all patients. We received approval from the UK Health Research Authority National Research Ethics Service Committee East Midlands, Nottingham 1 (ref. 14/EM/1126) and conducted all research in accordance with the Declaration of Helsinki.

### Imaging Acquisition

As previously reported, we scanned participants at 1.5T (Signa HDxt, General Electric, Milwaukee, WI) with a gradient-echo echo-planar imaging (GE-EPI) sequence (TR/TE = 3,000/45 ms, voxel size: 4 × 4 × 4 mm^3^) and neurovascular structural imaging protocol, including 3-D T1-weighted imaging; axial T2-weighted, axial fluid-attenuated inversion recovery; and axial gradient echo (Table 1A; Blair et al., 2020). We subsequently scanned a subset of patients at 3T (Siemens Verio, Siemens Healthcare, Erlangen, Germany) with a GE-EPI sequence (TR/TE = 3,000/30 ms, voxel size: 3 × 3 × 3 mm^3^) and comparable structural imaging protocol (Table 1B).

**TABLE 1:** MR sequence parameters used for **(A)** 1.5T and **(B)** 3T imaging protocols.

(A)	1.5T (Signa HDxt)				
Sequence	GE-EPI BOLD	3D IR-SPGR T1-w	T2-w Propeller	FLAIR	GRE
TR (ms)	3,000	9.6	7,000	8,000	900
TE (ms)	45	4.0	90	100	15
TI (ms)	−	500	−	2,000	−
Flip angle (°)	90	8	−	−	20
Field of view (cm)	25.6 × 25.6	25.6 × 25.6	24 × 24	24 × 24	24 × 24
Acquisition matrix	64 × 64	192 × 192		320 × 256	384 × 256
Slice thickness (mm)	4	1.3	4	4	4
Number of slices	36	160	36	36	36
Other	−	−	1.5 signal averages, matrix size 384	−	−

**(B)**	**3T (Siemens Verio)**				
**Sequence**	**GE-EPI BOLD**	**3D MPRAGE T1-w**	**T2-w BLADE**	**FLAIR BLADE**	**GRE**

TR (ms)	3,000	2,300	11,400	9,100	600
TE (ms)	30	2.98	120	125	10
TI (ms)	−	1,100	−	2,512	−
Flip angle (°)	90	9	−	130	15
Field of view (cm)	19.2 × 19.2	25.6 × 25.6	24 × 24	24 × 24	24 × 24
Acquisition matrix	64 × 64	256 × 256	384 × 384	256 × 256	320 × 256
Slice thickness (mm)	3	1	3	3	3
Number of slices	48	256	48	48	48
Other	−	−	−	−	−

**GE-EPI, gradient-echo echo-planar imaging; IR-SPGR, inversion recovery spoiled gradient echo; MPRAGE, magnetization prepared-rapid gradient echo; FLAIR, fluid-attenuated inversion recovery; GRE, gradient recalled echo.**

**FIGURE 1:**
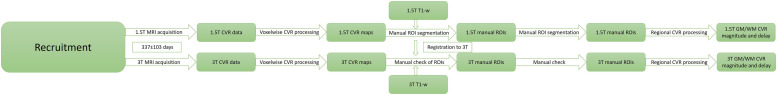
Schematic diagram of the study design and key analysis steps.

The full protocol for the CVR experiment has been previously described (Thrippleton et al., 2018). A summary of the key steps is provided in Figure 1. In brief, we administered alternating blocks of 2 min of medical air and 3 min of medical air with CO_2_ at an elevated concentration of 6% (BOC Special Products, United Kingdom). Gases were administered via a disposable anesthetic mask while wearing a unidirectional breathing circuit (Intersurgical, Wokingham, United Kingdom). Vital signs [peripheral oxygen saturation, blood pressure, heart rate, end-tidal CO_2_ (ETCO_2_), and respiratory rate] were measured throughout using a CD-3A CO2 sensor (AEI Technologies, Pittsburgh, United States) at 20 Hz and MR patient monitors at 1 Hz (Millennia 3155A at 1.5T and Magnitude 3150 MRI at 3T; In vivo, Best, Netherlands).

### Processing

An experienced neuroradiologist assessed SVD features of WMH, lacunes, microbleeds, visible PVS, and brain atrophy, using visual ratings on the 1.5T scans, including Fazekas score for WMH, PVS and atrophy scores, and counts of lacunes and microbleeds, which were used to calculate the SVD score (Staals et al., 2014; Blair et al., 2020).

We converted the DICOM files through SPM8 (Wellcome Department of Imaging Neuroscience, London, United Kingdom) (Friston, 2007). We discarded dummy scans before aligning the remaining volumes to the mean BOLD image of each patient in SPM8. We coregistered the T1-w images to the T2-w space using rigid-body registration calculating the transformation between T2-w and mean BOLD image spaces using FLIRT (FMRIB Analysis Group, Oxford, United Kingdom) (Jenkinson et al., 2002). We calculated the WMH and intracranial volume (ICV) from the 1.5T scans as previously described (Blair et al., 2020).

We converted the CO_2_ data to ETCO_2_ by using in-house MATLAB (MathWorks, Inc., MA, United States) code to identify CO_2_ peaks as previously described (Thrippleton et al., 2018). We resampled the ETCO_2_ data to match the temporal resolution of the BOLD data prior to linear regression.

We applied linear regression using in-house Matlab code to determine region-wise CVR magnitude and delay as described in Thrippleton et al. (2018). Briefly, we performed multiple linear regression with the time-shifted ETCO_2_ profile and volume number (to adjust for signal drift) regressors and percentage signal change relative to baseline as the dependent variable. The regression model was evaluated for a range of time shifts, and the model with lowest sum-of-square residuals was selected. CVR magnitude was reported as the regression coefficient for the optimally time-shifted ETCO_2_; CVR delay was reported as the optimal ETCO_2_ time shift plus 4 s (to account for the travel time of exhaled gas from the breathing circuit to the CO_2_ sensor). Heart rate and respiratory rate were not included as nuisance regressors as the acquisition was not synchronized to the physiological data and given the potential for aliasing.

### Regions of Interest for CVR Assessment

Due to the limited voxelwise contrast-to-noise, we chose 14 regions of interest (ROIs) to sample WM (frontal, periventricular, posterior, and centrum semiovale) and subcortical GM (caudate head, putamen, thalamus) regions affected in SVD (Thrippleton et al., 2018). We manually drew the regions on the coregistered 1.5T T1-w image in FSLview (FMRIB Analysis Group, Oxford, United Kingdom) following an established protocol (Thrippleton et al., 2018) and transformed the masks into the 3T T2-w space using FLIRT before thresholding, binarizing, and manually correcting the masks as needed to avoid misclassification of tissue. Manually defined stroke masks were used to exclude lesions. We coregistered the voxelwise CVR magnitude maps into the T2-w image using FLIRT and manually excluded blooming around the large veins and venous sinuses based on the voxelwise CVR magnitude maps before registering the ROIs to the mean BOLD image and calculating CVR magnitude and delay in each region at 1.5T and 3T using FSLview. We fitted the CVR for each region separately before averaging the CVR magnitude and delay values in WM and subcortical GM to calculate representative values.

### Statistical Analysis

We applied linear mixed models using R (v3.6.3) to assess the effect of tissue type (subcortical GM/WM) on CVR magnitude and delay adjusting for field strength. We plotted WM, subcortical GM, and ROI-based CVR magnitude and delay using scatterplots overlaid with univariable linear regression lines. We performed Wilcoxon signed rank tests to assess differences at 1.5T vs. 3T. Finally, we applied separate multivariable linear regression models to explore associations of CVR magnitude at 1.5T or 3T with age, sex, systolic blood pressure, and key metrics of SVD burden (WMH volume, total Fazekas score, and SVD score) (Blair et al., 2020). For each model, we checked normality of residuals and homogeneity of variance using Q-Q plots, histograms, and plots of residuals vs. fitted values for each model and, consequently, log transformed the WMH volume. No corrections were applied for multiple comparisons (Rothman, 1990; Saville, 1990).

## Results

**FIGURE 2:**
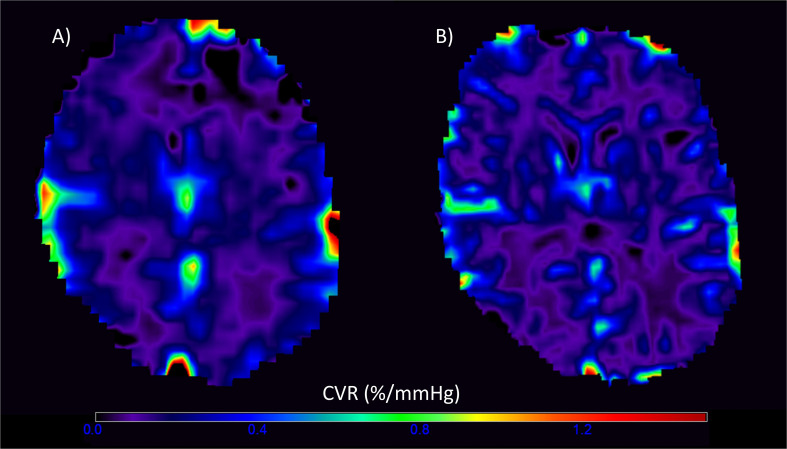
Representative voxelwise cerebrovascular reactivity (CVR) magnitude maps at 1.5T **(A)** and 3T **(B)** in the visit-specific T2-w space. The higher spatial resolution and CVR magnitude values are evident in the 3T image.

Of the 20 patients, 19 completed CVR scans at both 1.5T and 3T; one participant withdrew after experiencing a panic attack during the scan at 3T. One data set was excluded due to a poor-quality ETCO_2_ trace and persistent motion in the CVR scan at 3T, leaving 18 patients with complete data at both field strengths. The gas challenge otherwise obtained similar changes within each patient at both the 1.5T and 3T scans (*t*-test: *t* = 1.60, confidence interval (CI) = −0.34, 2.48, *p* = 0.127). Representative CVR magnitude images at 1.5T and 3T are shown in Figure 2.

The 18 patients had a mean age of 64 ± 7 (53–76) years; 14 (77.8%) were male, and 11 (61.1%) had a final stroke diagnosis of lacunar and seven (38.9%) of cortical subtype (Table 2). Imaging features of SVD were also common: eight (44%) had periventricular and six (33%) deep Fazekas score ≥2, and the median SVD score was two. The mean time between the 1.5T and 3T scans was 337 ± 103 (108–479) days.

**TABLE 2:** Demographic data of patients by SVD score (numerical variables: mean ± standard deviation (if normally distributed) or median (interquartile range); summary statistics: % of group (number); categorical variables presented as median).

	SVD score 0–1 (*n* = 8)	SVD score 2–4 (*n* = 10)	All patients (*n* = 18)
Age at visit	63 ± 7	65 ± 7	64 ± 7
Male	100% (8)	60% (6)	77.8% (14)
Diabetes	12.5% (1)	0% (0)	5.6% (1)
Hypertension	62.5% (5)	100% (10)	83.3% (15)
Systolic blood pressure	137 ± 12	140 ± 13	138 ± 12
Diastolic blood pressure	89 ± 5	89 ± 10	89 ± 8
WMH volume (ml), median (IQR)	6.55 (7.84)	14.11 (22.71)	11.26 (11.34)
WMH volume (% of ICV), median (IQR)	0.45 (0.53)	1.01 (1.22)	0.80 (0.74)
Deep Fazekas (median)	1	1.5	1
Periventricular Fazekas (median)	1	2	1
Total Fazekas score (median)	2	3.5	2
Lacunar subtype	50% (4)	70% (7)	61.1% (11)

**FIGURE 3:**
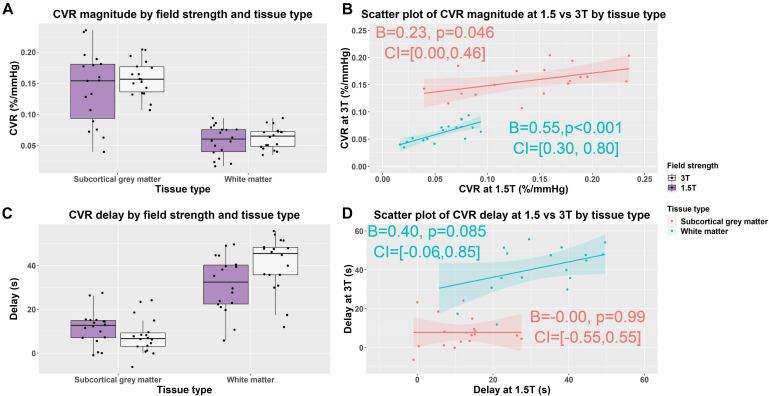
Box **(A,C)** and scatterplots **(B,D)** of mean cerebrovascular reactivity (CVR) magnitude and delay values in subcortical gray and white matter at 1.5 and 3T. Lines of best fit for the subcortical gray/white matter CVR magnitude and delay are shown separately in pink/blue. (B = effect size, CI = confidence interval, *p*-values uncorrected for multiple comparisons).

In subcortical GM compared with WM, independent of field strength, CVR magnitude was higher (1.5T: 0.142 vs. 0.057%/mmHg, 3T: 0.158 vs. 0.062%/mmHg; *B* = −0.09, CI = −0.10, −0.08, *p* < 0.001) and delay less (1.5T: 12 vs. 31 s, 3T: 8 vs. 41 s; *B* = 26, CI = 22, 31, *p* < 0.001) (Figures 3A,C).

### WM and Subcortical GM CVR Magnitude and Delay at 1.5T and 3T

We found no material difference in subcortical GM and WM CVR magnitude between 1.5 and 3T using Wilcoxon signed rank tests: subcortical GM (1.5T vs. 3T: 0.142 vs. 0.158%/mmHg; *r* = −0.30, CI: −0.044, 0.016, *p* = 0.212); WM (3 vs. 1.5T: 0.057 vs. 0.062%/mmHg; *r* = −0.33, CI: −0.013, 0.003, *p* = 0.167) (Figure 3A and Supplementary Table 1).

At 3T, delay tended to be shorter in subcortical GM (1.5 vs. 3T: 8 vs. 12 s; *r* = 0.33, CI: −2, 9, *p* = 0.164) and was longer in WM (1.5 vs. 3T: 31 vs. 41 s; *r* = −0.59, CI: −16, −2, *p* = 0.010) than at 1.5T (Figure 3C and Supplementary Table 1).

Scatterplots showed that the distribution of CVR magnitude values were narrower at 3T than 1.5T in both subcortical GM (CVR magnitude range at 3T: 0.107, 0.204; at 1.5T: 0.040, 0.235) and WM (CVR magnitude range at 3T: 0.035, 0.093; at 1.5T: 0.016, 0.094) (Figure 3B and Supplementary Table 1). Univariable linear regressions showed patients with higher CVR at 3T had higher CVR at 1.5T in both subcortical GM (*B* = 0.23, CI: 0, 0.46, *p* = 0.046) and WMr (*B* = 0.55, CI: 0.30, 0.80, *p* < 0.01).

The distribution of CVR delay values was similar at 3T and 1.5T in both subcortical GM (CVR delay range at 3T: −6, 24; at 1.5T: −1, 28) and WM (CVR delay at 3T: 12, 56; at 1.5T: 6, 50) (Figure 3D and Supplementary Table 1). Patients with longer delays at 3T tended to have longer delays at 1.5T in WM (*B* = 0.40, CI: −0.06, 0.85, *p* = 0.085), but not subcortical GM using univariable linear regressions.

### Regional Differences in CVR Magnitude and Delay

**FIGURE 4:**
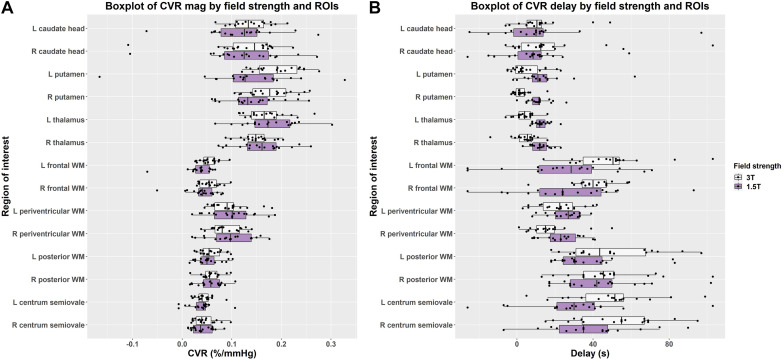
Boxplots comparing of **(A)** CVR magnitude (mag) and **(B)** delay values in subcortical gray and white matter regions at 1.5 and 3T (L = left, R = right).

In individual regions, the scatter of CVR magnitude values was mostly, but not always, larger at 1.5T than 3T though there was no obvious pattern for delay values (Figure 4 and Supplementary Table 1). Although regional CVR magnitude values were generally comparable at 3T and 1.5T, delay at 3T tended to be longer in WM and shorter in subcortical GM than at 1.5T.

### Effect of Field Strength on Associations With Demographic and SVD Imaging Variables

Although age, systolic blood pressure, and sex showed similar associations whether measured at 3T or 1.5T, the confidence intervals were broad, and none reached conventional significance (Supplementary Table 2).

We found CVR magnitude tended to be lower in patients with higher WMH volumes, Fazekas scores, and total SVD score at both 1.5 and 3T (Table 3 and Supplementary Figure 1).

**TABLE 3:** Associations of cerebrovascular reactivity (CVR) magnitude in subcortical gray and white matter at 1.5T and 3T with key SVD variables using multiple linear regression controlling for age, sex, and systolic blood pressure (*n* = 18).

	1.5T CVR		3T CVR	
	White matter	Subcortical gray matter	White matter	Subcortical gray matter
Log transformed WMH volume (norm to ICV)	*B* = −0.033 (−0.065, −0.001) *p* = 0.045	*B* = −0.055 (−0.144, 0.035) *p* = 0.211	*B* = −0.024 (−0.049, 0.002) *p* = 0.069	*B* = −0.013 (−0.061, 0.036) *p* = 0.578
Periventricular Fazekas score	*B* = −0.017 (−0.033, −0.002) *p* = 0.034	*B* = −0.030 (−0.075, 0.015) *p* = 0.173	*B* = −0.013 (−0.026, −0.001) *p* = 0.043	*B* = −0.011 (−0.035, 0.012) *p* = 0.320
Deep Fazekas score	*B* = −0.011 (−0.026, 0.005) *p* = 0.160	*B* = −0.015 (−0.057, 0.026) *p* = 0.444	*B* = −0.008 (−0.020, 0.005) *p* = 0.201	*B* = −0.004 (−0.025, 0.018) *p* = 0.727
Total Fazekas score	*B* = −0.007 (−0.015, 0.001) *p* = 0.073	*B* = −0.012 (−0.034, 0.011) *p* = 0.281	*B* = −0.005 (−0.012, 0.001) *p* = 0.093	*B* = −0.004 (−0.016, 0.008) *p* = 0.503
SVD score (high vs. low)	*B* = −0.004 (−0.031, 0.024) *p* = 0.782	*B* = −0.001 (−0.071, 0.070) *p* = 0.983	*B* = −0.013 (−0.033, 0.008) *p* = 0.197	*B* = −0.011 (−0.046, 0.025) *p* = 0.519

**Results presented as unstandardized B-values (effect size, change in CVR in %/mmHg per unit change in the independent variable), confidence interval and p-value without multiple comparison correction. Small vessel disease (SVD) score dichotomized as high, >1, and low, ≤1.**

## Discussion

We measured CVR magnitude and delay at 1.5 and 3T in a well-defined SVD cohort considering summary measures for averaged subcortical GM, WM, and 14 ROI-based measurements. CVR magnitude was similar, though more variable, at 1.5T vs. 3T in subcortical GM and WM. Delay was shorter at 3T in subcortical GM and longer at 1.5T in WM. Independent of field strength, we found CVR magnitude was higher and delay shorter in subcortical GM than WM, reflecting previous work (Thomas et al., 2014; Thrippleton et al., 2018).

### Effect of Field Strength on CVR Magnitude

Studies investigating the effect of field strength on CVR measurements using BOLD are limited. CVR magnitude was found to be higher at 3T than 1.5T using fixed inhalation hypercapnia (Triantafyllou et al., 2011) and breath-hold paradigms (Peng et al., 2020). Similarly, *R_2_^∗^*-derived CVR magnitude was higher at 7T than 3T using a targeted breathing ETCO_2_ paradigm (Driver et al., 2010). By contrast, we found that CVR magnitude did not differ materially between 3T and 1.5T though trending higher at 3T. However, there are several technical and methodological differences. Peng et al. (2020) used a breath-hold challenge that depends on compliance reported percentage rather than absolute changes (Tancredi and Hoge, 2013) and did not adjust for ETCO_2_, which can affect repeatability (Bright and Murphy, 2013). Additionally, they analyzed the signal in cortical GM, which may be more prone to vascular contamination than the subcortical GM regions considered in this work. BOLD CVR is known to be affected by echo time (Triantafyllou et al., 2011) and signal-to-noise ratio (SNR) (Triantafyllou et al., 2005), which can improve sensitivity and specificity to parenchymal response, particularly at 3T. However, the sequences used for this comparison were not independently optimized to maximize sensitivity to CVR-induced changes in the BOLD signal unlike in Triantafyllou et al. (2011). There are also benefits to lower field strengths as 1.5T scanners can show less geometric distortion and signal dropout due to susceptibility effects than at higher field strengths, affecting comparability in regions close to air–tissue boundaries, including the sinuses and base of the skull (Krüger et al., 2001). Blooming artifacts around large veins are also more severe at 3T (Chen et al., 2010).

### Differences in Delay

We found that average delay was shorter at 3T in subcortical GM and at 1.5T in WM. Although primarily a biological quantity, which would not be expected to vary with field strength, delay is challenging to measure (Thrippleton et al., 2018). We define delay as the optimal shift between ETCO_2_ and the BOLD signal, but due to the low SNR in WM (Thomas et al., 2014), particularly at 1.5T, delay values may approach a random distribution over the permitted range. Hence, delay measurements at 3T may better reflect underlying delay values. Although longer delays at 3T tended to be associated with longer delays at 1.5T in WM, there was no obvious association in subcortical GM. However, univariable linear regression is vulnerable to outliers, especially at extreme values, and although the range of delays was similar at both field strengths, the distribution was tighter and trended lower at 3T. The BOLD sequences acquired at 1.5T and 3T also differed in voxel size (4 × 4 × 4 vs. 3 × 3 × 3 mm^3^); therefore, differing levels of partial volume effect may influence the delay measurements. Overall, differences in delay values should be interpreted cautiously, and direct validation work in isolated vessel preparations or preclinical models to validate the influence of lower SNR and partial volume effect on delay measurements would be beneficial (Stringer et al., 2020).

### Regional Differences

Within the individual ROIs, we found generally similar results to the averaged values: mean CVR magnitude was comparable though marginally higher at 3T vs. 1.5T; delay in subcortical GM regions tended to be shorter at 3T than 1.5T; delay in WM regions was typically longer at 3T than at 1.5T. Although region-based measurements improve SNR relative to voxelwise measurements, they are still vulnerable to local BOLD signal differences, which must be accounted for in interpreting results. Venous blooming may affect CVR magnitude and delay values in the caudate head and thalamus; although we excluded voxels for which prominent vessels were visible in the ROIs, less prominent vessels were retained (Thrippleton et al., 2018). Smaller regions, including the caudate head, may be less stable given the small size. Meanwhile, in periventricular WM, CVR magnitude and delay may be altered by nearby venous structures (Nonaka et al., 2003), enhancing signal changes during the CVR paradigm. The higher spatial resolution of the BOLD sequence at 3T vs. 1.5T may allow more accurate identification of tissue boundaries and exclusion of contaminating large vascular structures while also reduce partial volume effect, which may contribute to reduced variance at 3T (Thrippleton et al., 2018).

### Associations Between CVR Magnitude, Demographic, and Other SVD Imaging Variables

Although CVR magnitude tended to be lower in patients who were older, male, and had higher blood pressure at both 1.5T and 3T, none of the associations reached conventional statistical significance, likely due to lack of power in this subset of the main study. Nevertheless the direction of effect was broadly consistent with previous work showing lower CVR in older patients (Hund-Georgiadis et al., 2003; Conijn et al., 2012; Gauthier et al., 2015; Blair et al., 2020), males than females (Kastrup et al., 1997; Cupini et al., 2001; Tallon et al., 2020), and in patients with higher blood pressure (Conijn et al., 2012; Haight et al., 2015).

We found that subcortical GM and WM CVR tended to be lower in patients with higher WMH volume, Fazekas (deep, periventricular, and total), and SVD score at both 3T and 1.5T, replicating previously published findings in a larger cohort (of which these patients formed a subgroup) scanned at 1.5T (Blair et al., 2020). Effect sizes were generally similar at 1.5T and 3T, but tended to be marginally higher at 1.5T than 3T for all associations except SVD score, which may be attributable to the small sample size and wider distribution of CVR magnitude values at 1.5T than 3T.

### Strength and Limitations

It was impractical to randomize scan ordering; therefore, habituation effects or time between scans may have influenced the results. Although the sample size was higher than for previously reported comparisons between CVR measurements at 3T and 1.5T (*n* = 9 and 4) (Triantafyllou et al., 2011; Peng et al., 2020), it was lower than some SVD studies using CVR (Blair et al., 2016, 2020). The exploratory multiple linear regression analyses may, therefore, have insufficient statistical power to detect more subtle effects though the results were consistent with previous work (Blair et al., 2020). Larger sample sizes and inclusion of other populations would be beneficial and, along with preclinical validation, may aid the development and wider applicability of CVR. The wider applicability of this comparison is also limited by the specific imaging protocols used though the sequences were appropriate for measuring CVR. We acquired CVR scans with a higher spatial resolution at 3T to mitigate higher blooming effects at 3T, which may also contribute to lower variance in the CVR magnitude. Approaches to sampling tissue vary, including ROI analyses (Triantafyllou et al., 2011) and whole-brain tissue segmentation approaches (Driver et al., 2010; Peng et al., 2020). We manually drew anatomically well-defined ROIs to obtain representative tissue volumes, potentially reducing the partial volume effect at tissue boundaries, the influence of venous blooming (Thrippleton et al., 2018), and atrophy (Catchlove et al., 2018). However, this may affect comparability with some previous studies. Additional analyses considering further regions, including cortical GM, or using voxelwise analysis methods would be worthwhile. Lastly further methodological work is required to assess comparability and encourage greater consensus on best practice. Although the reported CVR magnitude and delay values are comparable to previously reported values (Thrippleton et al., 2018), wider comparisons are challenging due to heterogeneity in the acquisition and processing methods (Sleight et al., 2021). Additionally, we have not explored reproducibility of CVR measurements using fixed CO_2_ inhalation at 3T although we previously tested reproducibility and different durations of CO_2_ exposure at 1.5T (Thrippleton et al., 2018).

## Conclusion

We demonstrate similar CVR magnitude values at 3T and 1.5T, which showed comparable tissue differences and associations with certain key clinical variables. We found wider scatter of magnitude values at 1.5T than 3T. Although delay differed between 1.5T and 3T depending on the brain regions sampled, this may result from differences in SNR, partial volume effect, and blooming artifact. Measuring and interpreting CVR delay, therefore, remains challenging. Regional variability highlights the importance of optimizing the tissue sampling strategy when designing studies.

## Data Availability Statement

The datasets presented in this article are not readily available because the data is stored on an internal database. However, the full individual anonymized dataset supporting the conclusions of this article, along with the study protocol, is available to bona fide researchers via the University of Edinburgh’s Cerebrovascular Diseases Database without undue reservation. Access requests should be submitted to JW along with a description of any planned analyses.

## Ethics Statement

The studies involving human participants were reviewed and approved by the UK Health Research Authority National Research Ethics Service Committee East Midlands, Nottingham 1. The patients/participants provided their written informed consent to participate in this study.

## Author Contributions

MS: data analysis, drafting, and revision of manuscript. GB: patient recruitment and revision of manuscript. YS: data acquisition, data analysis, and revision of manuscript. IH: data acquisition. DD: data analysis and revision of manuscript. FD: study design, development of the CVR procedure, and revision of manuscript. IM: study design, development of the CVR procedure and analysis techniques, data analysis, and revision of the manuscript. MT: data acquisition, development of the CVR procedure and analysis techniques, data analysis, and revision of the manuscript. JW: study conceptualization and design, development of the CVR procedure and analysis techniques, data analysis, and revision of the manuscript. All authors contributed to the article and approved the submitted version.

## Conflict of Interest

The authors declare that the research was conducted in the absence of any commercial or financial relationships that could be construed as a potential conflict of interest.
