# Supplementary material for: A Comparison of CVR Magnitude and Delay Assessed at 1.5 and 3T in Patients With Cerebral Small Vessel Disease
Source: Front Physiol. 2021 Jun 2;12:644837. doi: 10.3389/fphys.2021.644837 (PMC8207286; doi:10.3389/fphys.2021.644837)

**Supplemental Table 1**: Mean and standard deviation cerebrovascular reactivity (CVR) magnitude (%/mmHg) and CVR delay (s) with summary statistics from Wilcoxon signed rank tests comparing CVR values at 1.5 and 3T in each region of interest (*n*=18, r = effect size, calculated as $Z/\sqrt{n}$, CI = confidence interval, p values uncorrected for multiple comparisons, no data points were excluded as outliers).

| Region of interest | CVR (%/mmHg) | | | Delay (s) | | |
| --- | --- | --- | --- | --- | --- | --- |
|  | **1.5T** | **3T** | **Wilcoxon signed rank test** | **1.5T** | **3T** | **Wilcoxon signed rank test** |
| L caudate head | 0.119±0.074 | 0.137±0.039 | r=-0.23, CI: -0.055, 0.017, p=0.347 | 11±25 | 12±13 | r=-0.05, CI: -19, 5, p=0.856 |
| R caudate head | 0.124±0.082 | 0.127±0.072 | r=-0.16, CI: -0.043, 0.047, p=0.523 | 8±18 | 19±26 | r=-0.14, CI: -24, 5, p=0.558 |
| L putamen | 0.133±0.102 | 0.190±0.046 | r=-0.62, CI: -0.088, -0.016, p=0.007 | 14±14 | 5±9 | r=0.57, CI: 2, 15, p=0.013 |
| R putamen | 0.141±0.057 | 0.177±0.046 | r=-0.64, CI: -0.061, -0.014, p=0.005 | 11±6 | 2±4 | r=0.88, CI: 6, 11, p<0.001 |
| L thalamus | 0.177±0.057 | 0.169±0.042 | r=0.14, CI: -0.028, 0.041, p=0.580 | 13±4 | 4±5 | r=0.86, CI: 6, 12, p<0.001 |
| R thalamus | 0.161±0.052 | 0.148±0.030 | r=0.27, CI: -0.012, 0.039, p=0.265 | 12±5 | 4±6 | r=0.84, CI: 6, 11, p<0.001 |
| L frontal WM | 0.035±0.030 | 0.054±0.018 | r=-0.63, CI: -0.030, -0.006, p=0.006 | 26±28 | 49±21 | r=-0.69, CI: -37, -9, p=0.002 |
| R frontal WM | 0.040±0.031 | 0.055±0.020 | r=-0.47, CI: -0.025, -0.000, p=0.048 | 27±27 | 40±12 | r=-0.39, CI: -28, 2, p=0. 105 |
| L periventricular WM | 0.099±0.047 | 0.094±0.037 | r=0.16, CI: -0.013, 0.023, p=0.523 | 25±9 | 22±11 | r=0.39, CI: -1, 9, p=0.106 |
| R periventricular WM | 0.098±0.046 | 0.089±0.031 | r=0.20, CI: -0.009, 0.025, p=0.417 | 23±10 | 17±12 | r=0.49, CI: 1, 12, p=0.035 |
| L posterior WM | 0.053±0.025 | 0.058±0.022 | r=-0.34, CI: -0.014, 0.003, p=0.154 | 38±19 | 49±24 | r=-0.32, CI: -26, 6, p=0.189 |
| R posterior WM | 0.057±0.026 | 0.058±0.019 | r=-0.06, CI: -0.010, 0.008, p=0.832 | 43±22 | 48±22 | r=-0.07, CI: -17, 8, p=0.799 |
| L centrum semiovale | 0.037±0.026 | 0.044±0.018 | r=-0.20, CI: -0.018, 0.005, p=0.417 | 30±27 | 49±23 | r=-0.53, CI: -35, -5, p=0.021 |
| R centrum semiovale | 0.040±0.022 | 0.045±0.022 | r=-0.26, CI: -0.015, 0.005, p=0.284 | 39±23 | 52±22 | r=-0.53, CI: -26, -2, p=0.023 |
| Subcortical GM | 0.142±0.058 | 0.158±0.028 | r=-0.30, CI: -0.044, 0.016, p=0.212 | 12±8 | 8±8 | r=0.33, CI: -2, 9, p=0.164 |
| White matter | 0.057±0.024 | 0.062±0.018 | r=-0.33, CI: -0.013, 0.003, p=0.167 | 31±13 | 41±12 | r=-0.59, CI: -16, -2, p=0.010 |

**Supplemental Table 2**: Associations of cerebrovascular reactivity (CVR) magnitude in subcortical grey and white matter at 1.5T and 3T with age, blood pressure and sex using multiple linear regression controlling for the remaining variables and total Fazekas score as a representative disease variable (*n*=18). Results presented as unstandardised effect size (B), confidence interval, p value uncorrected for multiple comparisons.

|  | 1.5T CVR | | 3T CVR | |
| --- | --- | --- | --- | --- |
|  | White matter | Subcortical rey matter | White matter | Subcortical grey matter |
| Age (years) | B=-0.001  (-0.002, 0.001) p=0.298 | B=0.001  (-0.004, 0.005) p=0.793 | B=-0.001  (-0.002,0.000) p=0.287 | B=-0.000  (-0.003, 0.002) p=0.811 |
| Systolic blood pressure (mmHg) | B=-0.001  (-0.002, 0.000) p=0.081 | B=-0.002  (-0.004, 0.001) p=0.172 | B=-0.000  (-0.001, 0.000) p=0.373 | B=0.000  (-0.001, 0.002) p=0.876 |
| Sex (M=1, F=0) | B=-0.002  (-0.031, 0.027) p=0.876 | B=-0.002  (-0.082, 0.077) p=0.954 | B=-0.005  (-0.028, 0.018) p=0.654 | B=-0.021  (-0.063, 0.020) p=0.288 |

**Supplemental Figure 1**: Plots of residual cerebrovascular reactivity (CVR) controlling for age, sex, and systolic blood pressure against log transformed white matter hyperintensity volume (normalised to intracranial volume (ICV)), total Fazekas score and dichotomised SVD score (0=SVD score 0-1, 1=SVD score 2-4).

1.5T

3T

White matter Subcortical grey matter White matter Subcortical grey matter

Log transformed white matter hyperintensity volume (normalised to ICV)

Total Fazekas score

Dichotomised SVD score

(0=SVD score 0-1, 1=SVD score 2-4)

Residual CVR (%/mmHg)


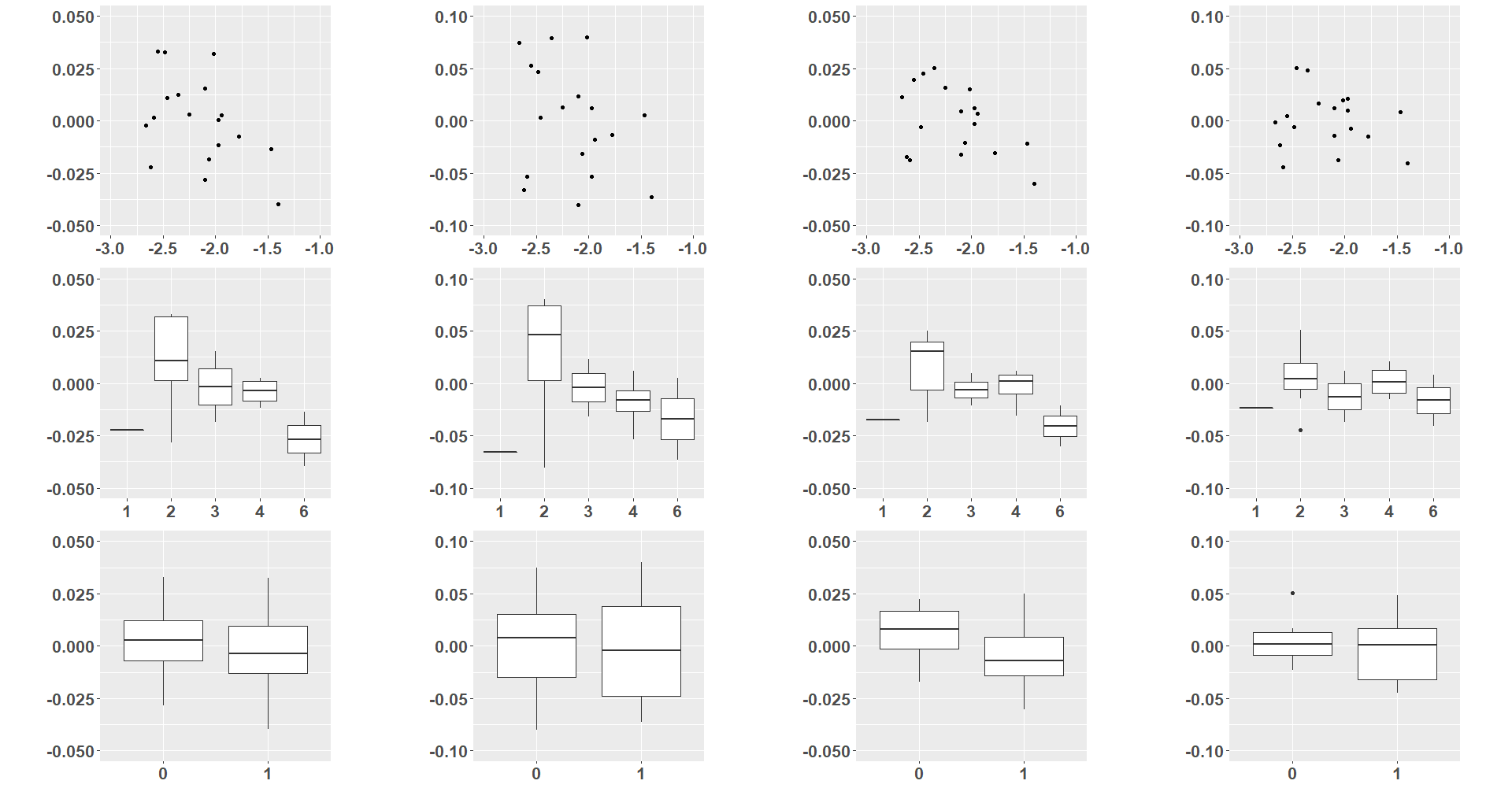

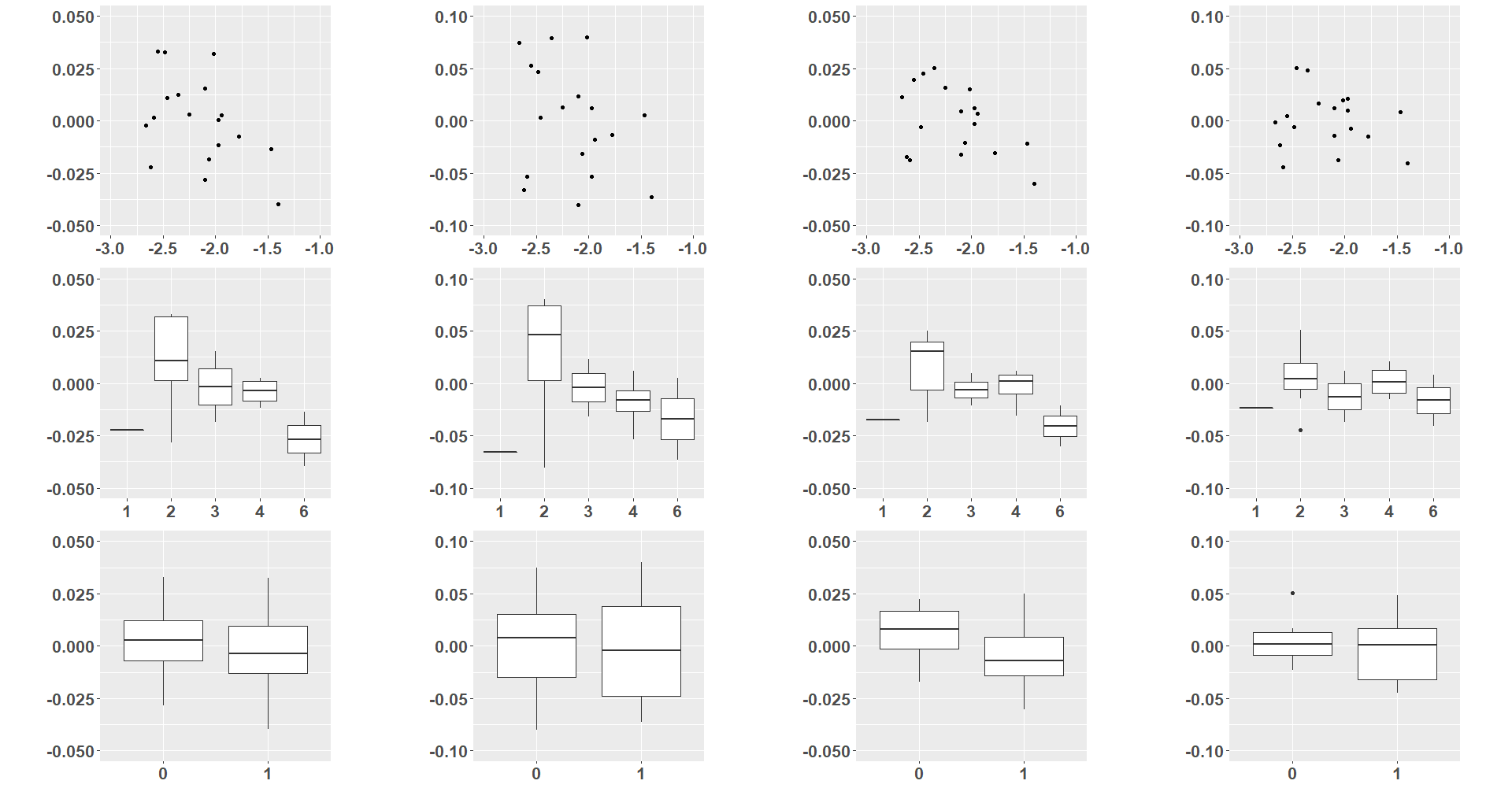

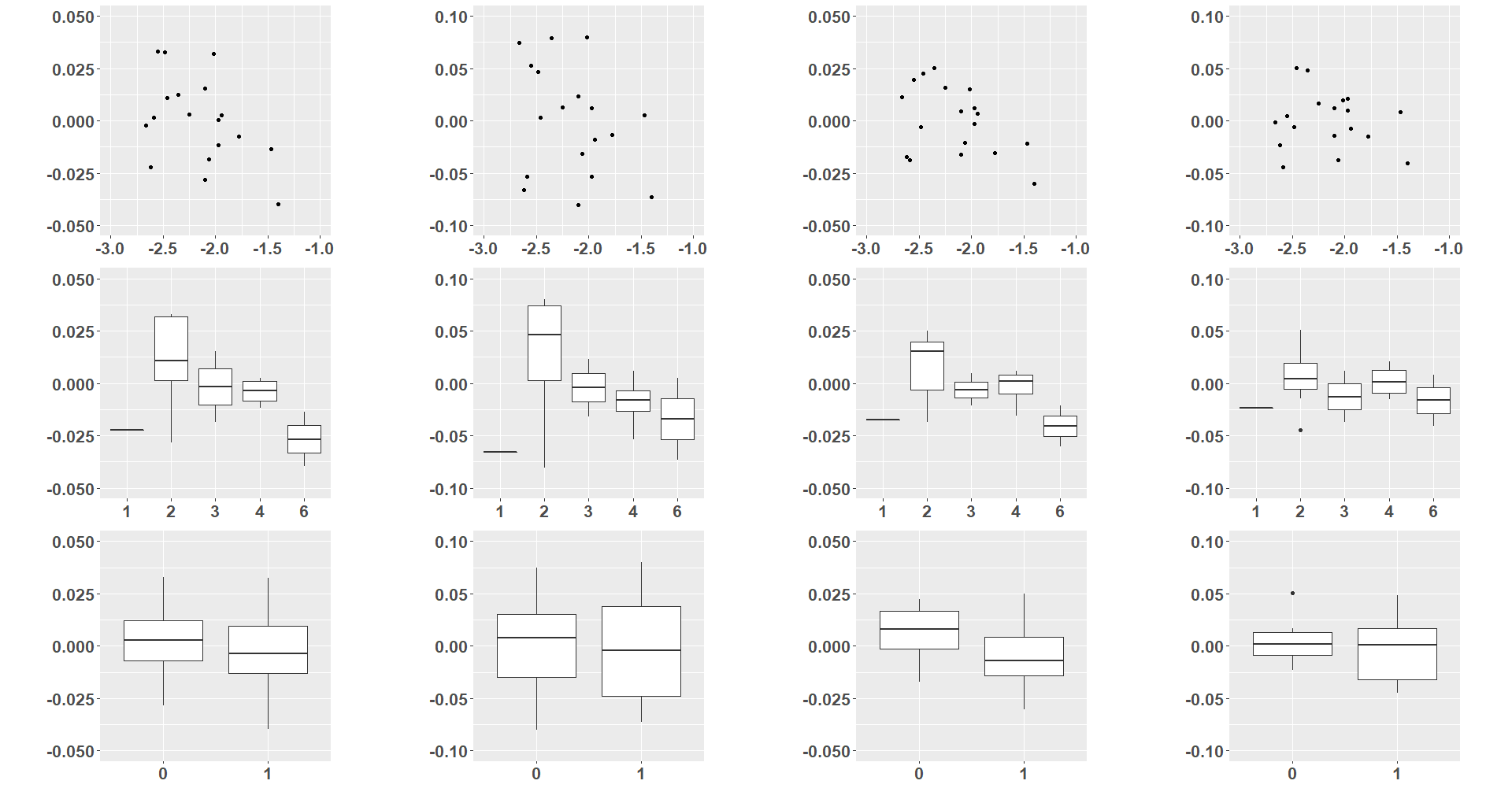

Supplement: Supplementary file 1 [file Data_Sheet_1.docx]
